# Characteristics of Terminated Medicare Advantage Contracts, 2011 to 2020

**DOI:** 10.1001/jamahealthforum.2022.3704

**Published:** 2022-10-21

**Authors:** David J. Meyers, Meehir N. Dixit, Amal N. Trivedi

**Affiliations:** 1 Department of Health Services, Policy, and Practice, Brown University School of Public Health, Providence, Rhode Island

## Abstract

This cross-sectional study characterizes Medicare Advantage contract terminations and identifies the characteristics of enrollees who may have been disproportionately affected by these events.

## Introduction

In the Medicare Advantage program (MA), the Centers for Medicare & Medicaid Services (CMS) pays private insurers capitated payments to cover enrollees’ health care. In 2022, MA accounted for 44% of all Medicare beneficiaries,^1^ and recent growth in MA enrollment has been greater among Black, Hispanic, and low-income beneficiaries.^2^

On an annual basis, CMS contracts with private insurers to provide Medicare services. These insurers may elect to terminate their MA contracts each year, or CMS can choose to terminate an insurer’s contract if it has consistently low performance. Terminations may be of notable concern to beneficiaries as they may disrupt their access to care and require a switch to a different MA plan or Traditional Medicare (TM) that may have vastly different benefits, out-of-pocket costs, physician networks, and medication formularies.^3,4^

Our objective was to characterize contract terminations and to identify the characteristics of enrollees who may have been disproportionately affected by these events.

## Methods

In this cross-sectional study, we used publicly available CMS plan crosswalk data to identify all MA contract terminations between 2011 and 2020. We only included contracts that ceased to exist in the following year necessitating an enrollee to change their enrollment and did not include consolidations in which 2 contracts merged and enrollees were assigned to the remaining contract.^5^ We linked these data to other publicly reported star ratings (an overall measure CMS uses for plan quality, ranging from 2 to 5 in 0.5 increments) and plan benefit data to compare plan characteristics. Using the 100% Medicare Master Beneficiary Summary file, we also calculated contract-level measures of enrollee characteristics, including the percentage of contract enrollees by race and ethnicity (defined based on administrative records and augmented with the Research Triangle Institute [RTI] race code) and the percentage of each contract’s enrollees that were dually enrolled in Medicaid. We compared characteristics using χ^2^, ANOVA, and *t* tests. A significance threshold of *P* < .05 with 2-tailed tests was used. Analyses were performed in Stata, version 17 (StataCorp LLC). This study met the STROBE reporting guideline and was determined to be exempt by the Brown University Institutional Review Board. Informed consent was waived because the data were deidentified.

## Results

This study included 935 unique MA contracts that were offered for at least 1 year between 2011 and 2020. Of these contracts, 170 (18.2%) were terminated, affecting 2.4% of MA beneficiaries (Table). Terminated contracts tended to have lower quality (mean star rating 3.1 vs 3.6, *P* < .001), tended to have $0 premiums (20.0% vs 13.2%, *P* = .001), and enrolled a greater proportion of Black (21.9% vs 14.3%, *P* < .001) beneficiaries. In all years but 2020, terminated contracts disproportionately enrolled Black beneficiaries compared with nonterminated contracts (Figure).

**Table.** Characteristics of Beneficiaries and Contracts by Termination Status^a^

Characteristic	Terminated	Not terminated	*P* value
Contracts, No. (%)	170 (18.2)	765 (81.8)	NA
Contract-years, No. (%)	170 (3.3)	5051 (96.7)	NA
Unique enrollees, No. (%)	769 696 (2.4)	31 026 103 (97.6)	NA
Plan type, No. (%)			
Health maintenance organization (HMO)	129 (75.9)	3658 (72.4)	.21
Preferred provider organization (PPO)	41 (24.1)	1393 (27.6)	
Mean (SD) premium, $	35.2 (38.5)	37.1 (36.2)	.38
Zero premium, No. (%)	34 (20.0)	668 (13.2)	.001
Average star rating, mean (SD)	3.1 (0.6)	3.6 (0.6)	<.001
Star rating, No. (%)			
2 to 2.5 stars	24 (14.1)	245 (4.9)	<.001
3 to 3.5 stars	51 (30.0)	1983 (39.3)	
4 to 4.5 stars	10 (5.9)	1324 (26.2)	
5 stars	0 (0.0)	106 (2.1)	
No rating	85 (50.0)	1393 (27.6)	
Enrollee race and ethnicity, mean %			
Asian/Pacific Islander	3.4	3.9	.44
Black	21.9	14.3	<.001
Hispanic	14.4	13.2	.49
Native American/Alaska Native	0.3	0.4	.41
White	58.4	66.4	<.001
Persons dually enrolled with Medicaid, mean %	36.1	31.8	.12
Year, No. (%)			
2011	10 (5.9)	512 (10.1)	<.001
2012	11 (6.5)	512 (10.1)	
2013	22 (12.9)	515 (10.2)	
2014	36 (21.2)	520 (10.3)	
2015	16 (9.4)	501 (9.9)	
2016	21 (12.4)	454 (9.0)	
2017	14 (8.2)	445 (8.8)	
2018	17 (10.0)	476 (9.4)	
2019	8 (4.7)	524 (10.4)	
2020	15 (8.8)	592 (11.7)	

Abbreviation: NA, not applicable.

^a^ All rows represent contract-level mean characteristics. Terminations in 2011 reflect contracts that existed in 2011, but no longer existed in 2012. Plan and enrollee characteristics are calculated based on the contract’s enrollment and characteristics in the year before it was terminated. *P* values are from χ^2^, ANOVA, or *t* tests depending on the variable format.

**Figure.**
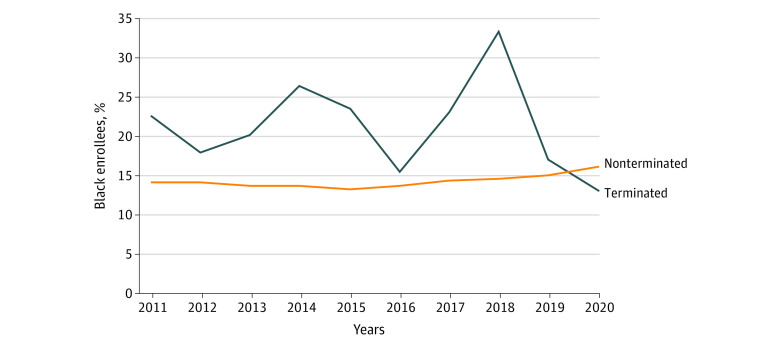
The Proportion of Black Enrollees in Terminated and Nonterminated Contracts The figure shows the average percentage of Black enrollees in contracts each year stratified by whether the contract was terminated or not. Terminations in 2011 represent contracts that existed in 2011 but no longer existed in 2012.

## Discussion

In this cross-sectional study from 2011 to 2020, almost 1 in 5 MA contracts were terminated, affecting more than 750 000 beneficiaries. These terminations may have had a disparate effect on Black beneficiaries who enrolled in terminated contracts at higher rates.

Although prior research has found that the termination of lower-quality contracts may be beneficial for some enrollees,^6^ terminations may still be disruptive for beneficiaries if they are associated with access to care or out-of-pocket spending. Although beneficiaries who do not select a new MA plan default into TM, they may be subject to substantial out-of-pocket spending if they lack supplemental coverage. Because MA contracts can selectively contract with a limited set of practitioners, an enrollee who needs to select a new contract may also experience disruptions in access to care. Given the potential disproportionate effect of terminations on Black beneficiaries, contract terminations may exacerbate disparities in access and continuity of care.

This study is limited in that we cannot distinguish contracts that were terminated by an insurer’s own decision from those that were terminated by CMS due to poor performance. However, both scenarios result in a potential disruption in an enrollee’s continuity of coverage.

As MA contracts become dominant payers in the Medicare program, it will be essential to understand how terminations affect the care and outcomes of enrollees. These findings demonstrate a substantial number of terminations from 2011 through 2020, and these terminations may have had a disproportionate effect on Black beneficiaries.
